# Vitamin A Absorption Determined in Rats Using a Plasma Isotope Ratio Method

**DOI:** 10.1093/jn/nxaa092

**Published:** 2020-04-09

**Authors:** Michael H Green, Joanne Balmer Green

**Affiliations:** Department of Nutritional Sciences, College of Health and Human Development, The Pennsylvania State University, University Park, PA, USA; Department of Nutritional Sciences, College of Health and Human Development, The Pennsylvania State University, University Park, PA, USA

**Keywords:** isotopes, isotope ratio method, rats, vitamin A, vitamin A absorption

## Abstract

**Background:**

Better methods are needed for determining vitamin A absorption efficiency.

**Objective:**

Our objective was to measure vitamin A absorption in rats by adapting a plasma isotope ratio method previously used to determine cholesterol absorption.

**Methods:**

Male Sprague-Dawley rats [*n* = 14; 340 ± 16 g (mean ± SD)] received an oral tracer dose of [^3^H]retinyl acetate in oil plus an intravenous dose of [^14^C]vitamin A–labeled lymph prepared in a donor rat that had received [^14^C]retinyl acetate intraduodenally. Blood samples were collected on days 1, 2, 3, 6, 9, and 12, and plasma was analyzed for ^3^H and ^14^C; vitamin A absorption was calculated for each sample as (fraction of oral dose/fraction of intravenous dose) × 100. Radioactivity was also measured in feces and urine collected as pools on days 3, 6, 9, and 12 and in liver and remaining carcass on day 12.

**Results:**

Vitamin A absorption calculated as the plasma isotope ratio was >100% on day 1, 78% ± 5% on day 6, 76% ± 5% on day 9, and 74% ± 5% on day 12; fitting the data to an exponential function plus a constant predicted an absorption of 75% by day 14. Recovery of the oral dose in feces (day 0 to day 6) was low (6.2% ± 0.84%, *n* = 10) and the mean isotope ratio in day 9–12 urine pool was lower than that in plasma.

**Conclusions:**

The plasma isotope ratio holds promise for estimating vitamin A absorption, but additional work is needed to determine how long studies need to be and if the doses should be administered simultaneously. For application of this method in humans, artificial chylomicrons labeled with a stable isotope of retinyl acetate could be used for the intravenous dose, with a different isotope required for the oral dose.

## Introduction

It is widely acknowledged that vitamin A absorption efficiency under various conditions has not yet been adequately defined in either animal models or humans (1, 2). Such information is crucial because absorption impacts recommendations for vitamin A intake and estimates of vitamin A status made using retinol isotope dilution; in addition, the estimate used for absorption influences compartmental model predictions related to whole-body vitamin A metabolism and stores. Vitamin A absorption has been measured directly in lymph of both rats (3) and humans (4) and indirectly in children using fecal balance methods (5, 6); however, the first technique is not routinely done and the second requires complete stool collection for at least several days after dosing.

Some years ago, we proposed (7) that a method described by Zilversmit (8) and Zilversmit and Hughes (9) for measuring cholesterol absorption in rats might be adapted to estimate vitamin A absorption. Zilversmit's idea was to measure the plasma isotope ratio after administering 1 cholesterol tracer orally and a different one intravenously. Once stabilized, the ratio of fraction of the oral dose in plasma divided by fraction of the intravenous dose in the same sample reflects absorption efficiency. Similar ratio methods have been used in humans to measure absorption of calcium (10), zinc (11), and vitamin E (12) as well as carotenoid relative bioefficacy (13) and bioconversion of β-carotene to retinol (14). Here we describe in more detail the application of this approach for measuring vitamin A absorption efficiency in rats and discuss its potential applicability in humans.

## Methods

### Animals and diets

Rat experiments were done in 1995; all procedures were approved at that time by The Pennsylvania State University's Animal Care and Use Committee. Adult male Sprague-Dawley rats (240–260 g) were purchased from Harlan Teklad; rats were individually housed in an environmentally controlled room with a 12-h light cycle; water and food [AIN-93 G diet (15) with 2.44 nmol retinyl palmitate (Sigma Chemical Co.) added per gram to provide ∼49 nmol/d] were available at all times for 3 wk before and during absorption studies; 2 d before the studies started, rats were transferred to metabolic cages (Nalge Co.).

### Radioactive vitamin A doses

Radiolabeled [^3^H]retinyl acetate (>99% pure) and [^14^C]retinyl acetate (98% pure) were generously provided by Hoffmann-La Roche and were stored at −80°C for ∼2 mo before this study. All procedures were conducted under shaded natural light or yellow light to minimize photoisomerization of vitamin A. For the oral dose, ∼200 µCi [^3^H]retinyl acetate was solubilized in soybean oil (4 g) and stored overnight at room temperature under a nitrogen atmosphere. For the intravenous dose, [^14^C]vitamin A–labeled lymph was prepared as follows. The cisterna chyli and thoracic duct of an anesthetized donor rat were cannulated as previously described (16) and the catheter was exteriorized for lymph collection. A double-lumen catheter was secured in the duodenum for administration of labeled oil (see below) and for continuous infusion of sterile saline (2.4 mL/h). During a 2-d recovery period, the rat had access to the diet described in the preceding section, water, and a salt drink (4 g/L NaCl + 0.3 g/L KCl in water). Approximately 48 h after surgery, ∼75 μCi [^14^C]retinyl acetate in soybean oil (0.6 g) was infused into the duodenum over a 10-h period. Lymph samples of 1-h duration were collected at 4°C into tubes containing sodium citrate. Samples with maximum radioactivity/gram were pooled and stored overnight at 4°C under a nitrogen atmosphere.

### Absorption studies

Rats [*n* = 14; 340 ± 16 g (mean ± SD)], presumably in their normal overnight postprandial state, were anesthetized with methoxyflurane (Pitman Moore) between 0850 and 1030 h and intubated with an accurately weighed amount (∼0.14 g) of the tritiated vitamin A–labeled oil. Immediately thereafter, an external jugular vein was exposed by blunt dissection and an accurately weighed aliquot (∼1 g) of [^14^C]vitamin A–labeled lymph was administered intravenously. Anesthesia was removed and rats were placed in clean metabolic cages. Blood samples (∼0.3 mL) were collected from a caudal vein into tubes containing Na_2_EDTA at 1, 2, 3, 6, 9, and 12 d after dose administration; aliquots of plasma were frozen at −16°C under a nitrogen atmosphere for subsequent analysis of radioactivity. Excreta were collected as cumulative pools on days 3, 6, 9, and 12. At each of these times, rats were transferred to a clean metabolic cage; feces were removed, weighed, frozen, lyophilized, and frozen at −16 °C for subsequent analysis of radioactivity. Cages were rinsed twice with a small volume (∼10 mL) of ethanol:water (1:1), and the urine + rinses were weighed and placed in aliquots for liquid scintillation spectrometry. On day 12, rats were killed with carbon dioxide: blood was collected from the left cardiac ventricle; the liver was excised, weighed, frozen, lyophilized, and stored at −16°C for subsequent analysis; and carcasses were also frozen for later analysis.

### Analyses

Aliquots of plasma were extracted from 50% ethanol into hexane containing 5 μg/mL butylated hydroxytoluene, using a modification (17) of the method of Thompson et al. (18). Solvent-free extracts were analyzed for radioactivity using Ready Organic (Beckman Instruments) as scintillation solution. Replicate aliquots of the radioactive doses and of freeze-dried liver were saponified and lipids were extracted into hexane as described by Duncan et al. (19). Portions of the solvent-free extracts were analyzed for radioactivity and, in the case of the doses, other aliquots were analyzed for retinol content by HPLC as previously described (20). Carcasses were ground and lipid-soluble radioactivity in replicate aliquots was analyzed using the methods described by Adams et al. (21). Replicate aliquots of urine (∼1 g) were counted using Ready Safe (Beckman) as scintillation solution. Replicate aliquots of freeze-dried feces for 10 of the rats were extracted by the method of Håkansson and Ahlborg (22) and analyzed for radioactivity using Ready Organic. All analyses for radioactivity were done using a Model 3801 (Beckman) liquid scintillation spectrometer; samples were each counted twice to a 2-σ error of 1.5%.

### Descriptive statistics

Data are presented as means ± SDs.

## Results

### Animal outcome

Body weights were 340 ± 16 g (*n* = 14) at the time of isotope administration and 359 ± 14 g at the end of the study (day 12). As anticipated, rats had adequate vitamin A status based on plasma retinol concentrations (2.3 ± 0.30 µmol/L) and estimated liver vitamin A concentrations (2.2 ± 0.44 µmol or 0.23 ± 0.048 µmol/g). Recovery of administered [^14^C] in donor lymph was 66% over 10 h; the labeled lymph administered as the intravenous dose was equivalent to ∼19 min of lymph and it provided ∼1.7 µCi, ∼76 nmol retinol, and ∼13 mg triglycerides; the [^3^H]vitamin A–labeled oral dose contained ∼9.8 µCi and ∼6 nmol retinol. The time between administration of the 2 doses averaged 4.8 min (range: 3–9 min) for the 14 rats.

### Vitamin A absorption based on plasma isotope ratios

Data on vitamin A absorption, calculated as the fraction of the orally administered isotope ([^3^H]) in plasma divided by the fraction of the [^14^C]-labeled lymph administered intravenously × 100, are shown in **Table 1** for the 6 sampling times. Values were >100% on day 1 as the intravenous pulse dose was rapidly cleared from plasma, presumably within minutes, whereas the oral dose was slowly absorbed over the course of several hours. By day 6, values were stabilizing, showing a much slower decline so that, on days 6, 9, and 12, mean absorptions were 78%, 75%, and 74%, respectively.

**TABLE 1:** Percentage of vitamin A absorption in rats determined by a plasma isotope ratio and asymptote fit^1^

Rat	Day 1	Day 2	Day 3	Day 6	Day 9	Day 12	Asymptote fit
1	329	167	112	80	76	74	76
2	152	121	92	73	71	70	70
3	206	137	105	85	83	81	82
4	132	112	90	77	75	74	74
5	295	162	115	83	80	78	80
6	210	137	99	76	73	72	73
7	173	125	98	83	81	80	81
8	153	109	83	68	65	63	64
9	141	118	97	82	81	81	80
10	168	129	95	72	69	68	68
11	142	109	92	78	76	74	75
12	178	126	97	78	74	72	74
13	199	122	93	75	76	74	75
14	167	119	92	79	78	76	77
Mean	189	128	97	78	76	74	75
SD	58	18	9	5	5	5	5

1 Values are observed data for (fraction of oral dose/fraction of intravenous dose) × 100 for 14 rats that received an oral dose of [^3^H]retinyl acetate in oil and an intravenous dose of lymph obtained from a donor rat that received [^14^C]retinyl acetate intraduodenally versus time after dose administration as well as the mean and SD. Also shown as asymptote fit are % absorption predicted by fitting the data to the equation *y*_1_*e*^(-^*^kt^*^)^ + *y*_2_.

**FIGURE 1:**
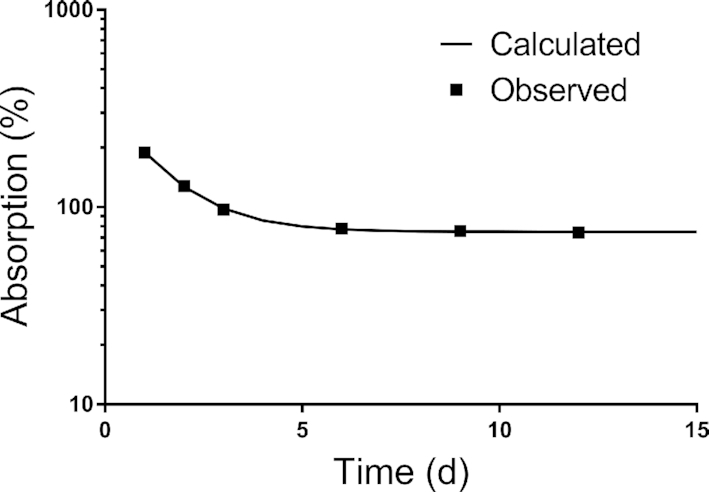
Vitamin A absorption determined by a plasma isotope ratio in rats. Shown are mean observed data versus time for (fraction of oral dose/fraction of intravenous dose) × 100 for 14 rats that received an oral dose of [^3^H]retinyl acetate in oil and an intravenous dose of lymph obtained from a donor rat that received [^14^C]retinyl acetate intraduodenally. The line shows the fit of the data to the equation *y*_1_*e*^(-^*^kt^*^)^ + *y*_2_ and indicates that the ratio reaches an asymptote plateau by 14 d after dose administration.

Since plots of the plasma isotope ratio over time indicated an exponential decline toward a constant value (which would presumably reflect the actual absorption efficiency), we fit the plasma isotope ratio data to a function described by an exponential decay plus a constant [i.e., *y*_1_*e*^(-^*^kt^*^)^ + *y*_2_], where *t* is time (days). As shown in **Figure 1** for the mean data, the ratio stabilized at a plateau value of 75% on day 14; absorption efficiencies for individual rats calculated using this asymptote method are shown in Table 1.

### Isotope recoveries in excreta, liver, and carcass

Only 6.2% ± 0.84% (*n* = 10) of the orally administered isotope and 4.0% ± 0.45% of the intravenous dose were recovered in feces from the time of dosing until day 6, with much smaller amounts in subsequent pools. Recoveries of the oral and intravenous doses in the day 0–3 urine pool were 3.7% ± 1.1% and 2.6% ± 0.50% (*n* = 14), respectively; values were lower in each subsequent pool, reaching 0.38% ± 0.15% and 0.63% ± 0.13% for days 9–12. Recovery of the orally administered tracer was 31% ± 5.1% (*n* = 14) in the liver on day 12 and 52% ± 7.7% for the intravenously administered isotope; recoveries in carcass were 4.6% ± 0.65% and 5.4% ± 0.89%, respectively; thus, 87% and 90% of the tracer found in liver + carcass was recovered in the liver for the orally and intravenously administered isotopes, respectively.

In addition to the plasma isotope ratio and asymptote plateau, we also estimated vitamin A absorption based on the loss of the tracers by calculating total tracer recovery in plasma + liver + carcass on day 12 and tracer loss as 1 minus tracer recovery for each isotope; then, loss difference = loss (oral tracer) − loss (intravenous tracer) and absorption = (1 − loss difference) × 100. Mean vitamin A absorption calculated by this method was 78% ± 3.9%, similar to the values of 74% determined using the plasma isotope ratio and 75% based on the asymptote plateau.

## Discussion

Here we used a simple plasma isotope ratio method to estimate vitamin A absorption efficiency in rats after simultaneous administration of 1 vitamin A label orally in oil and a different isotope, in a physiological carrier (donor lymph), intravenously; absorption measured 12 d later was 74% ± 5.3%. In comparison, Allen et al. (3) reported that vitamin A absorption in lymph-duct cannulated rats (*n* = 11) was 76% ± 15%; others have found lower values using that method (1). In the current study, we also calculated that absorption based on fitting the data to an exponential decay plus a constant was 75% ± 5.1% and, based on the fraction of the administered isotopes not recovered in plasma + liver + carcass over 12 d, absorption was 78%. The fact that estimated absorption is similar among these methods is encouraging. Our values are also very similar to the 76% reported by Aklamati et al. (6) based on isotope recovery in urine and feces after children received a small (6 mg) or larger (30 mg) amount of stable isotope–labeled vitamin A.

Plasma isotope ratio methods have been used to estimate absorption of several nutrients (see Introduction) as well as cholesterol (8, 9), and our current results indicate that the method holds promise for measuring vitamin A absorption. The method assumes that chylomicron-derived vitamin A is primarily taken up by the liver, that retinol is secreted into plasma bound to retinol-binding protein, and that plasma retinol exchanges with body pools of vitamin A before utilization. Since vitamin A in the intravenous dose was administered in chylomicrons, it represents 100% absorption, whereas the fraction of the oral dose that appears in chylomicrons is determined by the absorption efficiency. After sufficient time has elapsed, both tracers will have equilibrated with vitamin A in exchangeable body pools and the ratio in plasma stabilizes, reflecting absorption efficiency.

As applied to vitamin A, the isotope ratio method would require collection and analysis of just a single blood sample and thus it has advantages over other techniques such as AUC, in which multiple samples are collected over time, or fecal recovery, which is hampered by the possibilities of loss of label due to incomplete collection or bacterial degradation and by analytical difficulties. In the current studies, fecal recovery of the orally administered tracer was low (a cumulative 6% by 6 d). Although we recovered 11% ± 1.3% of the oral dose in excreta collected up to day 9, Håkansson and Ahlborg (22) recovered 23% of orally administered [^3^H]retinyl acetate in urine plus feces collected for 8 d after dosing in control rats. The reasons for this difference are unclear, especially since we used the same method for analysis of fecal radioactivity, but it is worth noting that rats in the 2 studies were fed very different diets. In contrast, our finding that the liver contained 87% of the oral dose recovered in liver + carcass on day 12 is comparable to the 85% reported by Håkansson and Ahlborg (22). We also found that 52% of the intravenously administered tracer was recovered in the liver on day 12 compared with ∼60% determined by Bausch and Rietz (23) 4 d after administration of an intravenous emulsion to rats with similar concentrations of liver vitamin A.

It has been reported that the ratio of an oral to intravenous dose in urine gives the same value for calcium absorption as does the ratio in plasma (24). In contrast, our results indicate that urine may not be useful for estimating vitamin A absorption using a dual-label method. Specifically, in the day 9–12 urine pool, the ratio of the 2 isotopes indicated a mean absorption of 60% (range: 46–99%), much lower than the mean based on the plasma isotope ratio and with a large range. However, the urine data proved valuable because they provide some experimental evidence for a hypothesis recently presented by Ford et al. (25). Specifically, their model describing the kinetics of orally administered [^13^C_10_]retinyl acetate in plasma of children includes a compartment into which newly absorbed retinol was directly transported for utilization before mixing with body pools. Our finding that ∼3% of both doses was recovered in urine in 3 d, compared with ∼0.7% at 6 d, indicates that there was utilization and urinary excretion of labeled vitamin A metabolites prior to mixing of the doses with vitamin A in stores. If mixing occurred prior to utilization, then recovery of label in urine at 3 d would have been much lower than that at 3–6 d. The data of Aklamati et al. (6) also indicate that there was utilization of labeled vitamin A administered orally to children prior to mixing of the dose with endogenous vitamin A.

To obtain reliable estimates of vitamin A absorption using the plasma isotope ratio, blood sampling must be done after the ratio has stabilized. In the case of cholesterol (8), that happened by 48 h after dosing; with a duration of 12 d for our vitamin A study, we felt confident that the ratio would reach a plateau. While our results (Table 1) indicate that the ratio was stabilizing by 12 d, it would have been ideal if we had collected additional samples. Based on a simple mathematical analysis, we conclude that the ratio would have reached a stable plateau by 14 d (Figure 1), but this needs to be confirmed in subsequent studies. In addition, a stable ratio might be obtained earlier if the intravenous dose were administered 2–3 h after the oral dose is given. In the current study, the relatively large mass of vitamin A administered as the intravenous dose (76 nmol in unprocessed donor lymph, compared with 6 nmol in the oral dose and an average intake of ∼50 nmol/d from the diet) might have perturbed the system slightly and therefore influenced stabilization of the isotope ratio in plasma. We recommend that, if further testing of this method is undertaken in rats, the vitamin A load in the intravenous dose should be minimized and its timing relative to the oral dose be considered. In addition, it might be useful to analyze both the retinyl ester and triglyceride content of the intravenous dose.

Our ultimate goal in proposing the dual isotope method for estimating vitamin A absorption was that it is a technique with potential applicability to determining absorption in humans. To date, no satisfactory method is available, but the need for such information is great among researchers who set recommendations for vitamin A intake, measure vitamin A status using retinol isotope dilution, or model whole-body vitamin A metabolism. Although the plasma ratio method holds promise for application in humans, an alternative to lymph, the physiological carrier for newly absorbed vitamin A, would be needed for the intravenous dose. In fact, Park et al. (26) have described the development and use of a tracer-labeled lipid emulsion to study the metabolism of chylomicron triglycerides in humans. These authors reported that the emulsion showed similar disappearance kinetics to intact chylomicrons. An analogous preparation could be developed and tested for vitamin A and then used, along with an oral dose labeled with a different stable isotope, in vitamin A absorption studies in humans.

## Notes

Support for these experiments was provided by USDA grant CRG 9500618; open access publication was funded by the Bill & Melinda Gates Foundation (project number OPP1115464).

Author disclosures: The authors report no conflicts of interest.

## References

[1] Blomhoff R, Green MH, Green JB, Berg T, Norum KR Vitamin A metabolism: new perspectives on absorption, transport and storage. Physiol Rev. 1991;71:951–90. 1924551 10.1152/physrev.1991.71.4.951

[2] International Atomic Energy Agency/Harvest-Plus/US Agency for International Development. Analysis of stable isotope data to estimate vitamin A body stores. Vienna: International Atomic Energy Agency; 2008.

[3] Allen LE, Green MH, Green JB Correspondence re: S.E. Dew et al., Effects of pharmacological retinoids on several vitamin A-metabolizing enzymes. Cancer Res. 1994;54:3319–20. 8205555

[4] Goodman DS, Blomstrand R, Werner B, Huang S, Shiratori T The intestinal absorption and metabolism of vitamin A and β-carotene in man. J Clin Invest. 1966;45:1615–23. 5925518 10.1172/JCI105468 PMC292843

[5] Sivakumar B, Reddy V Absorption of labelled vitamin A in children during infection. Br J Nutr. 1972;27:299–304. 5015249 10.1079/bjn19720094

[6] Aklamati EK, Mulenga M, Dueker SR, Buchholz BA, Peerson JM, Kafwembe E, Brown KH, Haskell MJ Accelerator mass spectrometry can be used to assess vitamin A metabolism quantitatively in boys in a community setting. J Nutr. 2010;140:1588–94. 20660280 10.3945/jn.110.125500 PMC3139233

[7] Green MH, Green JB Use of a plasma dual isotope ratio method to measure vitamin A absorption. FASEB J. 1997;11:A142.

[8] Zilversmit DB A single blood sample dual isotope method for the measurement of cholesterol absorption in rats. Proc Soc Exp Biol Med. 1972;140:862–5. 5039384 10.3181/00379727-140-36568

[9] Zilversmit DB, Hughes LB Validation of a dual-isotope plasma ratio method for measurement of cholesterol absorption in rats. J Lipid Res. 1974;15;465–73. 4472423

[10] van der Hee RM, Miret S, Slettenaar M, Duchateau SMJE, Rietveld AG, Wilkinson JE, Quail PJ, Berry MJ, Dainty JR, Teucher B et al. Calcium absorption from fortified ice cream formulations compared with calcium absorption from milk. J Am Diet Assoc. 2009;109:830–5. 19394469 10.1016/j.jada.2009.02.017 PMC2832736

[11] King JC, Donangelo CM, Woodhouse LR, Mertz SD, Shames DM, Viteri FE, Cheng Z, Welch RM Measuring iron and zinc bioavailability in humans. Food Nutr Bull. 2000;21:434–9.

[12] Traber MG, Leonard SW, Ebenuwa I, Violet P-C, Wang Y, Niyyati M, Padayatty S, Tu H, Courville A, Bernstein S et al. Vitamin E absorption and kinetics in healthy women, as modulated by food and by fat, studied using 2 deuterium-labeled α-tocopherols in a 3-phase crossover design. Am J Clin Nutr. 2019;110:1148–67. 31495886 10.1093/ajcn/nqz172 PMC6821549

[13] Ford JL, Green JB, Lietz G, Oxley A, Green MH A simple plasma retinol isotope ratio method for estimating β-carotene relative bioefficacy in humans is validated using model-based compartmental analysis. J Nutr. 2017;147:1806–14. 28747484 10.3945/jn.117.252361

[14] Ford JL, Green MH, Green JB, Oxley A, Lietz G Intestinal β-carotene bioconversion in humans is determined by a new single sample, plasma isotope ratio method and compared with traditional and modified area-under-the-curve methods. Arch Biochem Biophys. 2018;653:121–6. 29958897 10.1016/j.abb.2018.06.015 PMC6094152

[15] Reeves PG, Nielsen FH, Fahey GCJr. AIN-93 purified diets for laboratory rodents: final report of the American Institute of Nutrition ad hoc writing committee on the reformulation of the AIN-76A rodent diet. J Nutr. 1993;123:1939–51. 8229312 10.1093/jn/123.11.1939

[16] Sheehe DM, Green JB, Green MH Influence of dietary fat saturation on lipid absorption in the rat. Atherosclerosis. 1980;37:301–10. 7426103 10.1016/0021-9150(80)90016-7

[17] Green MH, Green JB, Lewis KC Variation in retinol utilization rate with vitamin A status in the rat. J Nutr. 1987;117:694–703. 3585518 10.1093/jn/117.4.694

[18] Thompson JN, Erdody P, Brien R, Murray TK Fluorometric determination of vitamin A in human blood and liver. Biochem Med. 1971;5:67–89. 5167174 10.1016/0006-2944(71)90076-7

[19] Duncan TE, Green JB, Green MH Liver vitamin A levels in rats are predicted by a modified isotope dilution technique. J Nutr. 1993;123:933–9. 8487104 10.1093/jn/123.5.933

[20] Green MH, Green JB Vitamin A intake and status influence retinol balance, utilization and dynamics in rats. J Nutr. 1994;124:2477–85. 16856330 10.1093/jn/124.12.477

[21] Adams WR, Smith JE, Green MH Effects of N-(4-hydroxyphenyl)retinamide on vitamin A metabolism in rats. Proc Soc Exp Biol Med. 1995;208:178–85. 7831350 10.3181/00379727-208-43849

[22] Håkansson H, Ahlborg UG The effect of 2,3,7,8-tetrachloro-dibenzo-*p*-dioxin (TCDD) on the uptake, distribution and excretion of a single oral dose of [11,12-^3^H]retinyl acetate and on the vitamin A status in the rat. J Nutr. 1985;115:759–71. 3998869 10.1093/jn/115.6.759

[23] Bausch J, Rietz P. Method for the assessment of vitamin A liver stores. Acta Vitamin Enzymol. 1977;31:99–112. 580694

[24] Moser-Veillon PB, Mangels AR, Vieira NE, Yergey AL, Patterson KY, Hill AD, Veillon C Calcium fractional absorption and metabolism assessed using stable isotopes differ between postpartum and never pregnant women. J Nutr. 2001;131:2295–9. 11533269 10.1093/jn/131.9.2295

[25] Ford JL, Green JB, Haskell MJ, Ahmad SM, Mazariegos Cordero DI, Oxley A, Engle-Stone E, Lietz G, Green MH Use of model-based compartmental analysis and a super-child design to study whole-body retinol kinetics and vitamin A total body stores in children from 3 lower-income countries. J Nutr. 2019;149:2065–72. 31535129 10.1093/jn/nxz225 PMC7004890

[26] Park Y, Grellner WJ, Harris WS, Miles JM A new method for the study of chylomicron kinetics in vivo. Am J Physiol Endocrinol Metab. 2000;279:E1258–63. 11093912 10.1152/ajpendo.2000.279.6.E1258

